# The minimal active domain of human salivary histatin 1 is efficacious in promoting acute skin wound healing

**DOI:** 10.1186/s40779-022-00398-9

**Published:** 2022-07-16

**Authors:** Xiao-Xuan Lei, Liu Hang-Hang Cheng, Hai-Yan Lin, Yu Yang, Yun-Yu Lu, Meng-Ru Pang, Yun-Qing Dong, Floris J. Bikker, Tymour Forouzanfar, Biao Cheng, Gang Wu

**Affiliations:** 1 Department of Oral and Maxillofacial Surgery/Pathology, Amsterdam UMC and Academic Center for Dentistry Amsterdam (ACTA), Vrije Universiteit Amsterdam, Amsterdam Movement Science, 1081HV Amsterdam, The Netherlands; 2 Department of Burn and Plastic Surgery, General Hospital of Southern Theater Command, Liuhua Road 111, Guangzhou, 510030 China; 3 Savaid Stomatology School, Hangzhou Medical College, Hangzhou, 310053 China; 4 Department of Plastic Surgery, the Third Affiliated Hospital of Guangzhou Medical University, Guangzhou, 510140 China; 5 Hangzhou Huibo Science and Technology Co. LTD, Xinjie Science Park, Hangzhou, 311217 China; 6 Department of Oral Biochemistry, Academic Center for Dentistry Amsterdam (ACTA), University of Amsterdam (UvA) and Vrije Universiteit Amsterdam (VU), 1081LA Amsterdam, The Netherlands; 7 Department of Oral Cell Biology, Academic Center for Dentistry Amsterdam (ACTA), University of Amsterdam (UvA) and Vrije Universiteit Amsterdam (VU), Gustav Mahlerlaan 3004, 1081LA Amsterdam, The Netherlands

**Keywords:** Histatin 1, Minimal active domain, Acute skin wound, Inflammatory response, Oxidative stress

Dear Editor,

The skin barrier can be impaired by acute skin wounds, which may lead to a series of complications. It is essential to accelerate wound healing and rapidly restore the structural integrity and functionality of skin. One of the promising bioactive agents is human salivary histatin 1 (Hst1), a 38-amino acid histidine-rich peptide that functions to maintain the homeostasis of oral mucosa with a cellular mechanism of promoting the adhesion, spreading, migration of epithelial cells and thus re-epithelialization [1]. In recent years, Hst1 has been shown to be effective against various skin-related cell types, such as fibroblasts, myo-fibroblasts, keratinocytes and endothelial cells. In our latest in-vivo study, Hst1 not only promotes angiogenesis, re-epithelialization and collagen production, but also suppresses inflammation, thereby significantly accelerating acute skin wound healing in mice [2]. All these studies show that Hst1 is a potent bioactive agent for accelerating acute skin wound healing.

However, in the field of synthetic therapeutic peptides, those with 15 or fewer amino acids are preferred due to high production/purification yields and low cost [3]. In previous studies, we have identified a 13-amino acid minimal active domain of Hst1 (Hst1-MAD, amino acid sequence: SHREFPFYGDYGS), which shows comparable efficacy in promoting the migration of epithelial cells [4] and skin dermal fibroblasts [5]. However, hitherto, the in-vivo effect of Hst1-MAD on acute wound healing has not been investigated. In this study, we aimed to systematically assess the therapeutic efficacy of Hst1-MAD on promoting acute skin wound healing in C57BL/6 mice. Twenty-seven mice were randomly divided into three treatment groups: 1) control (no treatment, negative control, *n* = 9); 2) 10 μmol/L Hst1 (positive control, *n* = 9); and 3) 1 μmol/L Hst1-MAD (*n* = 9). A round full-thickness wound of 1 cm-in-diameter was surgically created on the dorsal skin of each mouse. On days 3, 5 and 10 post-surgeries, the wounds were photographed and the healing percentage was gauged using ImageJ software. Thereafter, the wounds and surrounding tissues were retrieved, fixed and subjected to a series of histological, immunohistochemical and immunofluorescent staining and Western blotting to quantitatively assess angiogenesis, re-epithelialization, collagen expression, inflammatory response and oxidative stress.

Our results showed that the wound healing percentages in the 10 μmol/L Hst1 group (*P* = 0.049) and 1 μmol/L Hst1-MAD group (*P* = 0.014) were significantly higher than that of control group on day 3 post-surgery (Fig. 1a, b). On day 5 post-surgery, Hst1-MAD showed a slightly better healing efficacy than Hst1, but there is no statistical difference (Fig. 1b). The collagen expression level (*P* = 0.036, Fig. 1c), the surface area of CD31-positive blood vessels (*P* = 0.005, Fig. 1d) and the vascular endothelial growth factor (VEGF) expression level (*P* = 0.022, Fig. 1d) in the 1 μmol/L Hst1-MAD group were significantly higher than those in the control group. The expression intensities of two major epidermal tight proteins, claudin 1 (*P* = 0.039) and claudin 2 (*P* = 0.032) in the 1 μmol/L Hst1-MAD group were significantly higher than those in the control group (Fig. 1e). In addition, the expression level of claudin 2 in the 1 μmol/L Hst1-MAD group (*P* = 0.044, Fig. 1e) was significantly higher than that in the 10 μmol/L Hst1 group. However, 10 μmol/L Hst1 was significantly superior in the collagen expression level (*P* = 0.030, Fig. 1c) and the expression of claudin 1 (*P* = 0.031, Fig. 1e) than in the control group. Immunofluorescence double staining showed that the ratios of M2 (pro-wound healing) to M1 macrophages (pro-inflammatory) in the 1 μmol/L Hst1-MAD group (*P* = 0.011) and 10 μmol/L Hst1 group (*P* = 0.013) were significantly higher than that in the control group (Fig. 1f). Western blotting analysis revealed that 1 μmol/L Hst1-MAD significantly increased the expression level of NAD(P)H quinone oxidoreductase 1 (NQO1; antioxidative enzyme, *P* < 0.001), and reduced the expression levels of pro-inflammatory cytokines, such as tumor necrosis factor-α (TNF-α, *P* = 0.045), interleukin-6 (IL-6, *P* = 0.036) and macrophage inflammatory protein-1β (MIP-1β, *P* = 0.003; Fig. 1g).

**Fig. 1:**
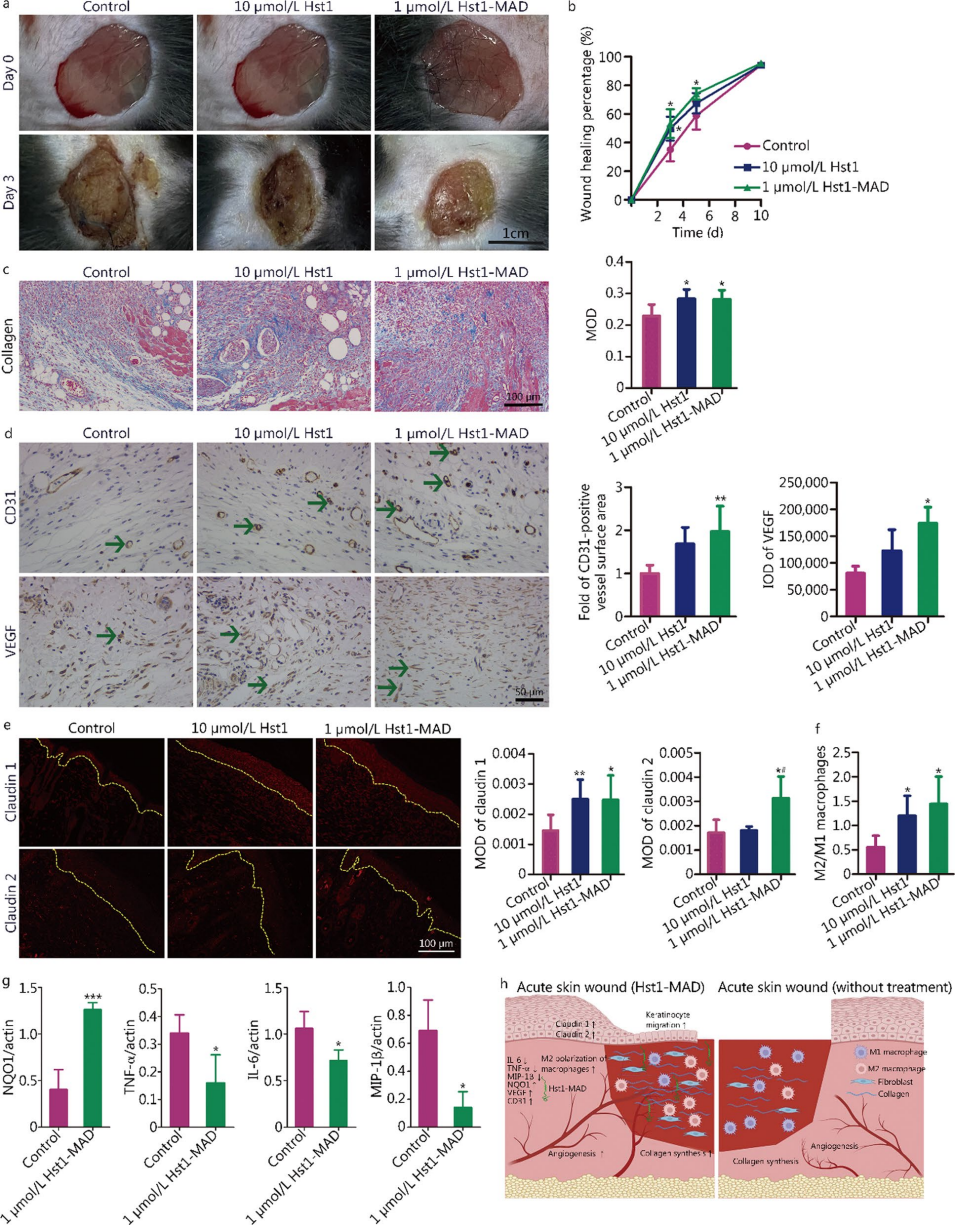
Effects of Hst1-MAD on acute wound healing. **a** Representative photographs of acute skin wounds in mice without treatment or treated with 10 μmol/L Hst or 1 μmol/L Hst1-MAD1 for 3 d (*n* = 9/group). **b** Wound healing percentages (%) in all groups on days 3, 5 and 10 post-surgeries. **c** Collagen fibers (blue stained) expressed in the newly formed dermal layers on day 5 post-surgery. The expression levels of collagen that were quantified using the function “count/size” of Image Pro plus and calculated using the formula: mean optical density (MOD) = integrated option density (IOD) sum/area sum. The sections were colored using Masson's trichrome staining. Scare bar = 100 μm (*n* = 6/group). **d** CD31-positive vessels and the positive expression of vascular endothelial growth factor (VEGF, green arrows) on day 10 post-surgery. The sections were immunohistochemically stained using corresponding antibodies to CD31 (GB13063; 1:300; Servicebio Inc., Boston, MA, USA) and VEGF (MA5-13182; 1:100; Thermo Fisher Scientific Co., CA, USA) and further counterstained with eosin. Scale bar = 50 μm. Angiogenesis were evaluated by the fold changes of the surface area of CD31-positive vessels and the IOD of VEGF (*n* = 6/group). **e** Expression of claudin 1 and claudin 2 (red color) in the newly formed epidermal layer (delineated in yellow curve) on day 10 post-surgery. The sections were immunofluorescently stained using respective antibodies to caludin 1 (37–4900; 1:100; Thermo Fisher Scientific Co., Shanghai, China) and claudin 2 (32–5600; 1:200; Thermo Fisher Scientific Co., Shanghai, China) (*n* = 6/group). **f** Ratio of M2 to M1 macrophages in the acute wound-surrounding tissues on day 5 post-surgery (*n* = 6/group). **g** Expression levels of endogenous antioxidant NAD(P)H quinone oxidoreductase1 (NQO1; ab28947; 1:1000; Abcam Trade Co., Shanghai, China) and a series of pro-inflammatory cytokines, such as tumor necrosis factor-α (TNF-α; ab6671; 1:1000; Abcam Trade Co., Shanghai, China), interleukin-6 (IL-6; ab9324; 1:1000; Abcam Trade Co., Shanghai, China) and macrophage inflammatory protein-1β (MIP-1β; C04131; 1:1000; Signalway Antibody Co., Nanjin, China). The expression of inflammatory factors of Hst1 has been detected in our previously published paper. Therefore, we did not evaluate it again in this study (*n* = 6/group). **h** Illustration of how Hst1-MAD enhanced the healing of acute skin wound. The diagram showing acute skin wound treated with Hst1-MAD (left) or without treatment (right). Data were presented as mean ± standard deviation. Statistical analyses in **b**, **c**, **d**, **e** and **f** were performed using one-way analysis of variance (ANOVA) with Bonferroni test as post-hoc comparison, in **g** were performed using *t*-test. ^*^P < 0.05, ^**^P  < 0.01, ^***^P < 0.001 vs. control group, ^#^*P* < 0.05 vs. 10 μmol/L Hst1 group

In conclusion, we found that 1 μmol/L Hst1-MAD could significantly promote the acute skin wound healing processes in-vivo by enhancing wound healing, re-epithelialization, collagen deposition, angiogenesis and the expression of tight junction proteins. Furthermore, Hst1-MAD could create a pro-wound healing microenvironment by not only significantly promoting the M2 polarization of macrophages and the expression of endogenous antioxidant, but also suppressing the expression of a series of pro-inflammatory cytokines (Fig. 1h). All these findings indicated a promising application potential in managing acute wound healing. The underlying molecular mechanisms remain to be investigated. Large animal studies are still needed to further confirm the potential for clinical application of Hst1-MAD.

## Data Availability

Not applicable.

## Abbreviations

Hst1: Histatin 1
Hst1-MAD: A 13-amino acid minimal active domain of Hst1
IL-6: Interleukin-6
MIP-1β: Macrophage inflammatory protein-1β
NQO1: NAD(P)H quinone oxidoreductase1
TNF-α: Tumor necrosis factor-α
VEGF: Vascular endothelial growth factor
